# Editorial: Components of the Language-Ready Brain

**DOI:** 10.3389/fpsyg.2016.00762

**Published:** 2016-05-23

**Authors:** Cedric Boeckx, Antonio Benítez-Burraco

**Affiliations:** ^1^Catalan Institute for Research and Advanced Studies (ICREA)/Department of General Linguistics, Universitat de BarcelonaBarcelona, Spain; ^2^Department of Philology and its Didactics, University of HuelvaHuelva, Spain

**Keywords:** language, language development, evolution, neurolinguistics, evolutionary biology

Our intention in putting together this volume was to exemplify and highlight new avenues of research in the language sciences concerning the neurobiology of language. We chose the term “language-ready brain” for our Research Topic, like we did for Boeckx and Benitez-Burraco, because we think it is high time to stress, on the one hand, the importance of a brain-based description of our species' linguistic capacity, and, on the other, the need to appreciate the crucial role culture plays in shaping the linguistic systems children acquire and adults use. In this sense, the focus of neurobiological investigations should not be “language,” but our learning biases and cognitive pre-dispositions toward language (i.e., “language-readiness”). Both brain and culture considerations ought to shape research at all levels of inquiry: phylogeny and ontogeny.

The contributions to this research topic break new grounds, by either revisiting long-standing issues (such as the role of Broca's region, the relevance of lateralization, the evolutionary origins of phonology, the role of basic cognitive and perceptive abilities in language acquisition, or the functions performed by language), or by examining closely issues that we are sure will rise to prominence in the near future (like the translational models of language processing into specific patterns of brain oscillations or the nature of the gene networks in which known “language genes” are found integrated). Taken together, the papers collected here shed light on language at the level of the genetics (van Rhijn and Vernes), brain connectivity (Murphy; Theofanopoulou), and physiology (Matchin and Hickok; Zaccarella and Friederici), cognition (de Boer; de Diego-Balaguer et al.), and behavior (Bouchard; Irurtzun; Reboul; Samuels).

In a fast-growing field like the language sciences, Research topics cannot hope to capture all relevant aspects of the field, but we hope that the present volume offers a snapshot that some of the most exciting research taking place today, sowing seeds for future investigations.

## Author contributions

Both authors wrote the editorial.

## Funding

Preparation of this work was supported by funds from the Spanish Ministry of Economy and Competitiveness (grants FFI2013-43823-P and FFI2014-61888-EXP), as well as funds from the Generalitat de Catalunya (2014-SGR-200).

### Conflict of interest statement

The authors declare that the research was conducted in the absence of any commercial or financial relationships that could be construed as a potential conflict of interest.

